# Supplementary material for: Experiences managing pregnant hospital staff members using an active management policy—A qualitative study
Source: PLoS One. 2021 Feb 26;16(2):e0247547. doi: 10.1371/journal.pone.0247547 (PMC7909656; doi:10.1371/journal.pone.0247547)
Supplement: S1 File — (PDF) [file pone.0247547.s001.pdf]

Interviewguide Danish version
(fokusgruppeinterview Aktiv Gravidpolitik)

Formål

At undersøge hvilke overvejelser og erfaringer ledere gør sig i ledelsen af gravide medarbejdere, samt beskrive deres erfaringer med anvendelse af en Aktiv Gravidpolitik (AGp)

Inden interviewet udfyldes kort spørgeskema, samt samtykkeerklæring

Indledning	**Velkommen (formål)**, I er inviteret fordi i er ledere og fordi I har deltaget i AGp…. Vi præsenterer os som jordemødre med uddannelse i forskning.<br>**Sikre anonymitet** : Det er vigtigt at understrege at I er sikret anonymitet, det betyder at data ikke bliver tilgængelig for andre end forskningsgruppen og at jeres identitet og arbejdssted ikke bliver offentliggjort.<br>**Præsentationsrunde** + navneskilte<br>**Fokusgruppeinterviewets form** tale/diskutere med hinanden<br><br>**Præsentation af moderator og sekretær** 'Vi er her for at lære af jer' (oplevelser og holdninger) der findes ingen rigtige svar<br>(B Halkier kap 3, s. 58)
Spørgeområde 1	**Den generelle oplevelse af at være leder for gravide medarbejdere**<br><br><u>Åbningsspørgsmål:</u> "fortæl lidt om de erfaringer I har gjort - med gravide medarbejdere i jeres tid som leder"<br><br>"Gør man sig som leder særlige overvejelser i ledelsen af gravide medarbejdere?"<br><br>"Hvad oplever I, som særligt vigtigt i samarbejdet med en gravid medarbejder (hvornår lykkes det)?"<br><br>"Er der tilstrækkelige muligheder for at tage hensyn til eller give en gravid medarbejder andre opgaver?"<br><br>(Det undrer mig at ingen af jer har nævnt…)hjælpesætning Mange ledere har talt om holdningen/tilgangen hos de gravide (at nogle VIL sygemeldes lige meget hvad)

Spørgeområde 2	**AGp´s anvendelse i praksis og vurdering af udbytte.**<br><br>"Oplevede I det som relevant at blive inviteret på seminar i AGp?<br><br>Har det ændret jeres tilgang til gravide medarbejdere i praksis?<br><br>"Hvilke udfordringer har I oplevet ved at skulle omsætte AGp til den kliniske dagligdag? " er der nogen?<br><br>"Hvordan vil I vurdere udbyttet (hvad har det betydet) at have deltaget i projektet og dermed også seminaret" uddyb gerne
Spørgeområde 3	**Forbedringstiltag**<br><br>"Hvad skal der til hvis opgaven med at forbedre trivslen for gravide medarbejdere og derved nedbringe sygefraværet, skal gøres lettere/bedre? (organisatorisk, lovgivning mm)"<br><br>Der har været tale om tre-parts samtaler (gravid, leder, fagperson) som en mulighed. Hvad er jeres tanker om det
Afslutning	Når I sådan lige tænker tilbage på hvad vi har talt om, er der så noget I har lyst til at tilføje, eller måske temaer vi ikke har været inde omkring?
