# Supplementary material for: Experiences managing pregnant hospital staff members using an active management policy—A qualitative study
Source: PLoS One. 2021 Feb 26;16(2):e0247547. doi: 10.1371/journal.pone.0247547 (PMC7909656; doi:10.1371/journal.pone.0247547)
Supplement: S2 File — (PDF) [file pone.0247547.s002.pdf]

Interview guide

Focus group interview; Experiences managing pregnant hospital staff members using an active management policy

Aim

To investigate the experiences and considerations of managers in relation to the management of pregnant employees and to describe the experiences they have obtained using an active pregnancy police

The informed consent form and the short questionnaire are filled out before the interview

introduction	**Welcome (purpose), "**you are invited because you are managers and because you have participated in active management policy project.<br>We introduce ourselves as midwives with experience in research**.**<br><br>**Securing anonymity: "**It is important to emphasize that you are guaranteed anonymity, this means that data will not be available to anyone other than the research group and that your identity and workplace will not be published.<br><br>**Presentation** presentation of the informants and of the scope of a Focus group interview talk and mutual discussions<br><br>**Presentation of the moderator and secretary '**We are here to learn from you' (experiences and attitudes) there are no right answers
Area of interest 1	**The general experience of being a manager for pregnant employees**<br><br>**Opening question:** "Tell us a little about the experiences you have had - with pregnant employees in your time as manager"<br><br>"Do you, as a manager, have special considerations in the management of pregnant employees?"<br><br>"What do you experience as particularly important in working with a pregnant employee (when do you feel like you have succeeded, in this role)?"<br><br>"Are there sufficient opportunities to allow for pregnant employees to work in their own pace or give them employee other tasks if necessary?"

Area of interest 2	**Active management policy in use and discussions of benefits**<br><br>“Did you find it relevant to be invited to a seminar in Active management policy for pregnant employees?”<br><br>“Has it changed your approach to pregnant employees in practice?”<br><br>“Did you experience any challenges using the policy in the clinical daily routine?”<br><br>"How would you describe the benefit (what has it meant to you) to have participated in the project and thus also the seminar?"
Area of interest 3	**Improvement suggestions**<br><br>“In our opinion, what will it take to improve the well-being of pregnant employees and thereby reducing unnecessary sick leave? (organizational, legislation, etc.)"<br><br>“Have been experience in three-party conversations (pregnant empolyee, manager, professional) do you consider this an option? What are your thoughts on that?”
End question	“If you just think back on what we've talked about, discussed today, is there anything you want to add, or maybe areas of interest we haven’t discussed?”
