# Supplementary material for: Experiences managing pregnant hospital staff members using an active management policy—A qualitative study
Source: PLoS One. 2021 Feb 26;16(2):e0247547. doi: 10.1371/journal.pone.0247547 (PMC7909656; doi:10.1371/journal.pone.0247547)
Supplement: S3 File — (PDF) [file pone.0247547.s003.pdf]

Content of the INTERVENTION

All managers, at the workunits allocated to intervention, were invited to participate in a ***three-hour seminar****

The three-hour seminars
The seminars were held at the workplaces and tutors were an occupational physician and a former hospital manager, who used an active pregnancy policy as a manager at an emergency department

Content of the seminar was:
- Knowledge about occupational risk factors in relation to pregnancy
- Introduction to the *Active Pregnancy policy concept (APp-concept)*
- Case-based work with the concept

Each manager received:
- A printed booklet about the *APp-concept*
- A printed booklet about occupational risk factors in relation to pregnancy
- A poster with the study name to display at their work units.

Managers in the intervention were given access to a Hotline, if they had questions about occupational factors in relation to pregnant employees and they were given username and password to www.aktivgravidpolitik.com, that held all seminar presentations, printed materials and detailed study information

The APp-concept
A positive, solution-oriented proactive approach, which consits of:

1. *A visible and acknowledged policy of pregnancy* at the work unit
2. *A congratulation letter* from the manager to the pregnant employee inviting her to the first meeting
3. *Planned meetings* during the pregnancy between the manager and the employee with an individual risk assessment and talk about worries and needs for job adjustments
4. *Job adjustments* when needed (fx changes in work schedules, different work tasks, extra small breaks)

* At two hospitals participation was mandatory, whereas particpation was voluntary in the other three hospitals

The Active management policy

Congratulation letter

The letter/mail is send/given when the manager is informed about the pregnancy. It contains:

1) Invitation to and date for the first meeting with the manager
2) A list over work assignments (risk assessment)
3) An overview of offers to pregnant employees at the workplace

Planned meetings

3 planned interviews during pregnancy, the first within 1-2 weeks after the information about pregnancy

1) Individual risk assessment (using the list of work assignments)
2) Discover any worries the pregnant employee might have
3) Investigate whether there is a need for job adjustments

At interview 2 and 3 new and ongoing worries, needs and adjustments are discussed and planned

Visible Pregnancy policy

Job adjustment

Examples of adjustments:

- Changes in work schedule (e.g. no early hours if problems with nausea in the morning, no night shifts, shorter shifts)
- Changes in work tasks (e.g. more administrative – sitting work)
- Extra small breaks
- A place to rest
